# Proteomics Study of the Synergistic Killing of Tigecycline in Combination With Aminoglycosides Against Carbapenem-Resistant *Klebsiella pneumoniae*

**DOI:** 10.3389/fcimb.2022.920761

**Published:** 2022-06-30

**Authors:** Xinqian Ma, Shining Fu, Yifan Wang, Lili Zhao, Wenyi Yu, Yukun He, Wentao Ni, Zhancheng Gao

**Affiliations:** Department of Pulmonary and Critical Care Medicine, Peking University People’s Hospital, Beijing, China

**Keywords:** *Klebsiella pneumoniae*, tigecycline, aminoglycoside, combination therapy, hypersensitivity

## Abstract

Co-administration of antibiotics with synergistic effects is one method to combat carbapenem-resistant organisms. Although the synergistic effects of tigecycline combined with aminoglycosides against carbapenem-resistant Klebsiella pneumoniae (CRKP) have been demonstrated *in vitro* and in animal models, the underlying mechanism remains elusive. Here we used proteomics analysis to assess the short-term bacterial responses to tigecycline and aminoglycosides alone or in combination. Emergence of tigecycline resistance during treatment and the susceptibility of tigecycline-resistant strains to aminoglycosides was further evaluated. The proteomic responses to tigecycline and aminoglycosides were divergent in monotherapy, with proteomic alterations to combination therapy dominated by tigecycline. Adaptive responses to tigecycline were associated with the upregulation of oxidative phosphorylation and translation-related proteins. These responses might confer CRKP hypersensitivity towards aminoglycosides by increasing the drug uptake and binding targets. Meanwhile, tigecycline might perturb adaptive responses to aminoglycosides through inhibition of heat shock response. Tigecycline-resistant strains could be isolated within 24 h exposure even in strains without heteroresistance, and the sensitivity to aminoglycosides significantly increased in resistant strains. Overall, these findings demonstrated that adaption to tigecycline in CRKP was a double-edged sword associated with the synergistic killing in tigecycline–aminoglycoside combination. Evolutionary hypersensitivity can provide novel insight into the mechanisms of antibiotic synergistic effects.

## Introduction

Carbapenem-resistant organisms (CROs) make once-treatable bacterial infections life-threatening and undermine the achievements of modern medicine. Carbapenem-resistant *Enterobacteriales*, especially carbapenem-resistant *Klebsiella pneumoniae* (CRKP), are recognized as among the highest priority pathogens by the WHO (Tacconelli et al., 2018). Infections caused by CRKP are associated with high morbidity and mortality (Wang et al., 2022), which significantly increase clinical and economic burdens. CRKP are generally multidrug resistant and only susceptible to a few antibiotics, including tigecycline, colistin, cefiderocol and one or more aminoglycosides (Durante-Mangoni et al., 2019). However, the clinical efficacy of antibiotic monotherapy is not optimal, and CRKP resistance to last-line antibiotics is increasing due to frequent use and selection pressure (Grundmann et al., 2017).

The combination of antibiotics offers a promising strategy to address the widespread emergence of CROs (Karaiskos et al., 2017; Maryam et al., 2019). Drug interactions can be classified as additive, synergistic, or antagonistic depending on their combined effect being identical, greater, or less than predicted on the basis of drug individual effects (Sullivan et al., 2020). Synergistic combination therapies can kill microbes more efficiently and may slow the evolution of resistance against CROs (Davis et al., 2021). The mode of action of antibiotic combination therapy is generally drug- and species-specific and without uniform rules (Maryam and Khan, 2016; Maryam and Khan, 2017; Brochado et al., 2018; Sullivan et al., 2020). Our previous studies demonstrated the synergistic effects of tigecycline combined with aminoglycosides (amikacin or gentamicin) against CRKP using *in vitro* and animal models (Ni et al., 2021), suggesting co-administration of these agents as a promising approach for treating CRKP infections.

The underlying mechanisms of the synergistic effect between tigecycline and aminoglycosides remain elusive, and previous studies even suggest possible antagonism of the antibiotic combinations: 1) both tigecycline and aminoglycosides act on the decoding center of the bacterial ribosome small subunit (Lin et al., 2018), and co-administration may result in competitive binding with ribosomal targets (Griffith et al., 1965; Garrett et al., 1970; Parfait et al., 1981; Di Giambattista et al., 1986); 2) tigecycline exerts antibacterial effects by inhibiting bacterial growth, whereas aminoglycosides can induce cell death. Generally, cell death from most bactericidal antibiotics requires cellular respiration and metabolic flux, which can be prevented by bacteriostatic drugs (Ocampo et al., 2014; Lobritz et al., 2015; Stokes et al., 2019). The dissimilarity between observation and postulation indicates a gap in our current understanding of the synergistic mechanisms in combinations of tigecycline and aminoglycosides. Deciphering the mechanisms of synergistic effects between these two drugs will help us understand the combined effects of ribosome-targeting antibiotics on protein synthesis, cellular processes, and stress responses in bacteria.

Considering that tigecycline and aminoglycosides both interfere with protein synthesis (Lin et al., 2018), proteomics would be superior in portraying universal protein variations in antibiotic combination therapies. Therefore, in this study, we first assessed the short-term proteomic response of CRKP exposed to tigecycline and aminoglycosides alone or in combination, and explored the underlying mechanisms of the synergistic effects of this combination. Then we evaluated the emergence of tigecycline resistance during treatment and the susceptibility of tigecycline-resistant strains to aminoglycosides.

## Materials and Methods

### Strains, Antibiotics, and Reagents

We collected 100 nonduplicate CRKP clinical isolates from four tertiary hospitals in Beijing, China, from June 2014 to December 2018. Strain K-28 was collected from a patient (65-years old) with a bloodstream infection (Ni et al., 2021). All strains were identified using the automated VITEK 2 system (bioMérieux, Marcy-l’E´toile, France). The MICs for K-28 against tigecycline, amikacin, and gentamicin are 0.5 mg/L, 2 mg/L, and 0.5 mg/L, respectively.

Amikacin and gentamicin were obtained from the National Institute for the Control of Pharmaceutical and Biological Products (NICPBP, Beijing, China). Tigecycline was purchased from Bide Pharmatech (Shanghai, China). Cation-adjusted Mueller–Hinton broth (CAMHB) and Mueller–Hinton agar (MHA) were obtained from Difco (Franklin Lakes, New Jersey, USA). The minimum inhibitory concentrations (MICs) were determined by standard broth microdilution methods and disk-diffusion methods according to the recommendations of the Clinical and Laboratory Standards Institute (CLSI) (CLSI, 2020). The susceptibility results for tigecycline were interpreted according to European Committee on Antimicrobial Susceptibility Testing (EUCAST) guidelines (tigecycline susceptible, ≤1 mg/L; tigecycline resistant, >2 mg/L) (EUCAST, 2018).

### *In Vitro* Synergy Tests by Checkerboard Method

We used checkerboard method to assess antagonism and synergy between drug combinations in strain K-28, as previously described (Ni et al., 2021). Briefly, drug concentrations were adjusted from 4× MIC to 1/128× MIC values according to the MIC tests. Overnight culture bacterial suspension was diluted to ~1×10^6^ CFU/mL, and 100 μL of bacterial suspension was added to 96-well microdilution plates along with 50 μL of tigecycline and 50 μL of amikacin or gentamicin. The microplate was then incubated aerobically at 37°C for 20 h, after which the optical density at 600 nm (OD_600_) was measured. The fractional inhibitory concentration index (FICI) was used to assess antibiotic interactions [FICI = (MIC _drug A in combination_/MIC _drug A alone_) + (MIC _drug B in combination_/MIC _drug B alone_)].

### *In Vitro* Synergy Tests by Time-Kill Assay

Overnight cultures of *K. pneumoniae* were adjusted to 0.5 McFarland and diluted 1:10 using freshly prepared CAMHB. Diluted bacterial cultures (~1×10^7^ CFU/mL) were mixed with fresh CAMHB containing a single antibiotic or a pair of antibiotics at a concentration of 1× MIC and incubated under shaking at 37°C. Samples were collected from the tube at various time points (0, 2, 4, 6, 12, and 24 h) after inoculation and serially diluted. The diluted samples (100 μL) were then plated on blank MHA plates and freshly prepared MHA plates with 2 mg/L and 4 mg/L tigecycline to select the resistant clones. The number of bacterial colonies was counted after 24 h of incubation at 37°C. Synergy was defined as a reduction of ≥2 log_10_ CFU/mL in the group receiving combination treatment as compared with the most active drug alone.

### *In Vivo* Synergy Tests Using the Rat Tissue Cage Infection Model

The animal study was performed as previously described (Ni et al., 2021). Fully anesthetized rats were subcutaneously implanted with a plastic tube containing holes (4 mL). Rats were intraperitoneally administered penicillin (400,000 IU/kg/day) for 3 days and allowed to recover for 2 weeks. Approximately 1-2×10^10^ CFU bacteria were subsequently injected into the tube. Successful tissue cage infection was defined as a bacterial density >1×10^9^ CFU/mL after 2 days of infection. Clinically recommended doses of antibiotics were administered after dosage conversion, which were 5mg/kg q12h of tigecycline with corresponding loading doses, and 100mg/kg and 35mg/kg of amikacin and gentamicin, respectively. Then 100 µL of tissue cage fluid was extracted for viable count on days 1 and 5 after treatment. The animal study was approved by the Institutional Animal Care and Use Committee of Peking University People’s Hospital (2020PHE032).

### Bacterial Culture for the Proteomics Experiment

Three to four colonies of K-28 grown on MHA plates were chosen and grown overnight, inoculated in 20 mL CAMHB, and incubated with shaking (180 rpm) at 37°C. The overnight culture was then adjusted to 0.5 McFarland and diluted 1:100 with CAMHB. The diluted culture was grown to an OD_600_ of 0.3 ± 0.02 and transferred to six 250-mL conical flasks. The solutions of antibiotics were then added to five of six flasks with final concentrations of MICs for each antibiotics, including monotherapy and combination therapy. The remaining flask acted as a drug-free control. Proteomics and related studies were conducted using a relatively higher bacterial inoculum (5×10^7^ CFU/mL) than the time-kill assays in order to prevent excessive bacterial killing. The flasks were further incubated under shaking at 37°C for 2 h. To account for inherent random variation, three biological replicates were prepared independently on different days for each condition.

### Proteomics Analysis

Total proteins were extracted from bacterial samples and were quantified using a Pierce BCA Protein Assay Kit BCA (Thermo Fisher Scientific, Waltham, MA, USA). Proteins were reduced and alkylated, then digested by trypsin. Firstly, data-dependent acquisition (DDA) mode was used to build a spectra library. Briefly, equal amounts of trypsin-digested peptides of each sample were pooled together, dried under vacuum and resuspended. Then the mixture was fractionated by Vanquish Flex ultra-high-performance LC (Thermo Fisher Scientific, Waltham, MA, USA) with an ACQUITY UPLC BEH C18 column (1.7 µm, 2.1 mm × 150 mm; Waters Corp., Milford, MA, USA). Next, the peptides were redissolved and analyzed by an on-line nanoelectrospray Q Exactive HF-X quadrupole Orbitrap mass spectrometer (Thermo Fisher Scientific, Waltham, MA, USA) coupled with an EASY-nLC 1200 system (Thermo Fisher Scientific, Waltham, MA, USA). The individual samples of each group were then analyzed in data-independent acquisition (DIA) mode.

The DDA data were analyzed by ProteomeDiscoverer (Version 2.4; Thermo Fisher Scientific, Waltham, MA, USA) with the default settings, and the DIA data were analyzed using Spectronaut (Version 14.0; Biognosys AG, Schlieren, Switzerland). Differentially expressed proteins (DEPs) were identified according to a fold change (FC) >1.2 or <0.83 and an adjusted *P* < 0.05. DEPs were further used for Gene Ontology (GO) and Kyoto Encyclopedia of Genes and Genomes (KEGG) enrichment analyses, and *P* values were adjusted by Benjamini-Hochberg method. Detailed information of proteomics analysis is provided in **Text S1**.

### Population Analysis Profiling Assay

Population analysis profiling assays were performed as previously described (Band et al., 2019). Briefly, solid agar plates were prepared with tigecycline at seven concentrations [0-, 0.125-, 0.25-, 0.5-, 1.0-, 2.0-, and 4.0-fold that of the breakpoint (1 mg/L), as defined in the EUCAST guidelines (EUCAST, 2018). CRKPs without tigecycline resistance were grown overnight in CAMHB from a single colony, and serial microdilutions were inoculated at each concentration of tigecycline on MHA plates, with colonies enumerated after incubation at 37°C. Isolates were classified as heteroresistant if the number of colonies growing at 2.0- or 4.0-fold that of the breakpoint concentration was at least 0.0001% (1/10^6^) of those growing on antibiotic-free plates.

### qRT-PCR Analysis

Total bacterial RNA was extracted using the RNAprep pure cell/bacteria kit (Tiangen, Beijing China) according to manufacturer instructions. cDNA was synthesized using the TIANScript cDNA kit (Tiangen, Beijing China), and the expression of *ramA* and *acrB* was quantified using a 7500 real-time PCR system (Applied Biosystems, Foster City, CA, USA). The specific primers are listed in **Table S1**. Housekeeping gene *rrsE* was used as an internal reference. Relative quantification of target genes (*ramA* and *acrB*) was performed with the 2^−ΔΔCt^ method.

### Statistical Analysis

Normally-distributed variables were compared using Student’s *t* test or analysis of variance, and abnormally-distributed continuous variables were compared using the Mann–Whitney *U* test or the Kruskal–Wallis H test. All statistical tests were two-sided, and statistical significance was defined as a *P* < 0.05 or an FDR < 0.05.

### Data Availability

The proteomics data have been uploaded to the ProteomeXchange Consortium *via* the iProX partner repository (Ma et al., 2019) (PXD031030).

## Results

**Figure 1:**
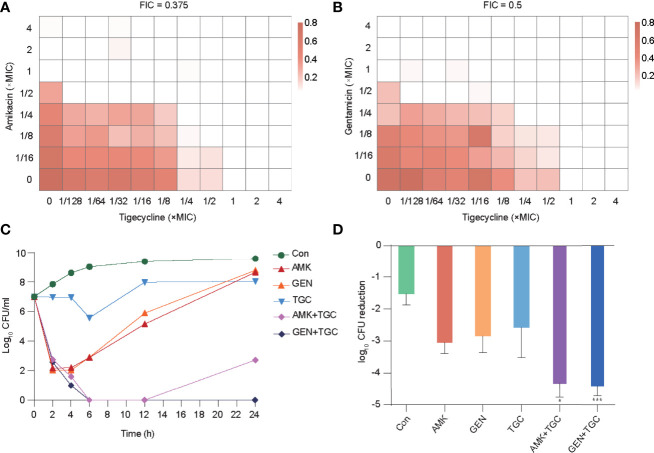
*In vitro* and *in vivo* synergistic effects of tigecycline combined with aminoglycosides against K-28. **(A, B)** The combined inhibitory effects of 1/128× to 4× MIC tigecycline and either **(A)** 1/16× to 4× MIC amikacin or **(B)** 1/16× to 4× MIC gentamicin were tested using the checkerboard method. Bacterial growth is shown as a heatmap. **(C)** Time-kill curves of tigecycline or aminoglycosides alone or in combination at 1× MIC concentration. **(D)** *In vivo* antimicrobial efficacy of tigecycline combined with aminoglycosides in a rat tissue cage infection model. Combination therapy was compared with the most effective monotherapy. Error bars indicate SDs. *P < 0.05; ***P < 0.001. Con, control; TGC, tigecycline; AMK, amikacin; GEN, gentamicin.

Our previous studies demonstrated the *in vitro* and *in vivo* synergistic effects of tigecycline combined with aminoglycosides against CRKP (Ni et al., 2021). In the present study, we selected a representative strain (K-28) isolated from the blood of a patient with bloodstream infection for further analysis. K-28 is an ST11 KPC-producing *K. pneumoniae*, which is a highly prevalent clone in China (Zhang et al., 2017; Qin et al., 2020). *In vitro* synergistic effects were found in both tigecycline/amikacin and tigecycline/gentamicin combinations (**Figures 1A–C**) using both the checkboard and time-kill methods. In the rat tissue cage infection model, we observed a significantly greater reduction in CFUs with combination therapy, representing the *in vivo* synergistic efficacy (**Figure 1D**).

### Study Design and General Results of Proteome Analysis

**Figure 2:**
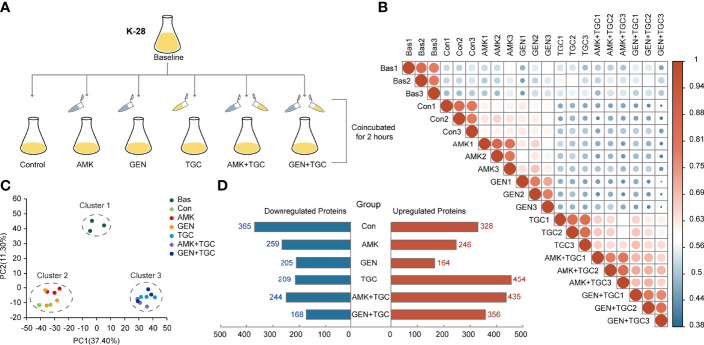
Study design and general results of proteome analysis. **(A)** Experimental design of the proteomics analysis. **(B)** Correlation plots of total proteome data from three biological replicates in different study groups. Colored circles represent Pearson’s correlation coefficients. **(C)** Principal component analysis score plots of total proteome data from three biological replicates from six study groups. **(D)** The number of DEPs in each study group. DEPs were defined as an FC >1.2 or FC <0.83 and FDR <0.05. Bas, baseline; Con, control; TGC, tigecycline; AMK, amikacin; GEN, gentamicin.

To determine the underlying mechanisms of the synergy between tigecycline and aminoglycosides against CRKP, we assessed the short-term proteomic responses of K. pneumoniae K-28. Strains during the exponential growth stage were collected as baselines and incubated with a single drug and drug combinations for 2 h, with strains incubated without antibiotics as controls (**Figure 2A**). Three biological replicates were used for each group. The DIA mass spectrometry-based proteomics was employed for protein quantification, and a total of 2,873 proteins were identified with a FDR <1% for protein and peptide identification, which covered about 50% of the expected total proteome. The inter- and intragroup similarities are shown in **Figure 2B**, with the high similarity among the three biological replicates indicating the robustness of the experiment. We identified three clusters in principal component analysis of the proteomics data, including cluster 1 (baseline group), cluster 2 (control group and strains treated with aminoglycosides), and cluster 3 (tigecycline monotherapy and combination therapies) (**Figure 2C**). **Figure 2D** shows the number of DEPs (FC >1.2 or <0.83 and FDR <0.05) in each group. Proteomic alterations were less with gentamicin monotherapy, and DEPs were mainly upregulated in tigecycline monotherapy and combination therapies.

### Distinct Proteomic Response Induced by Different Therapeutic Strategies

**Figure 3:**
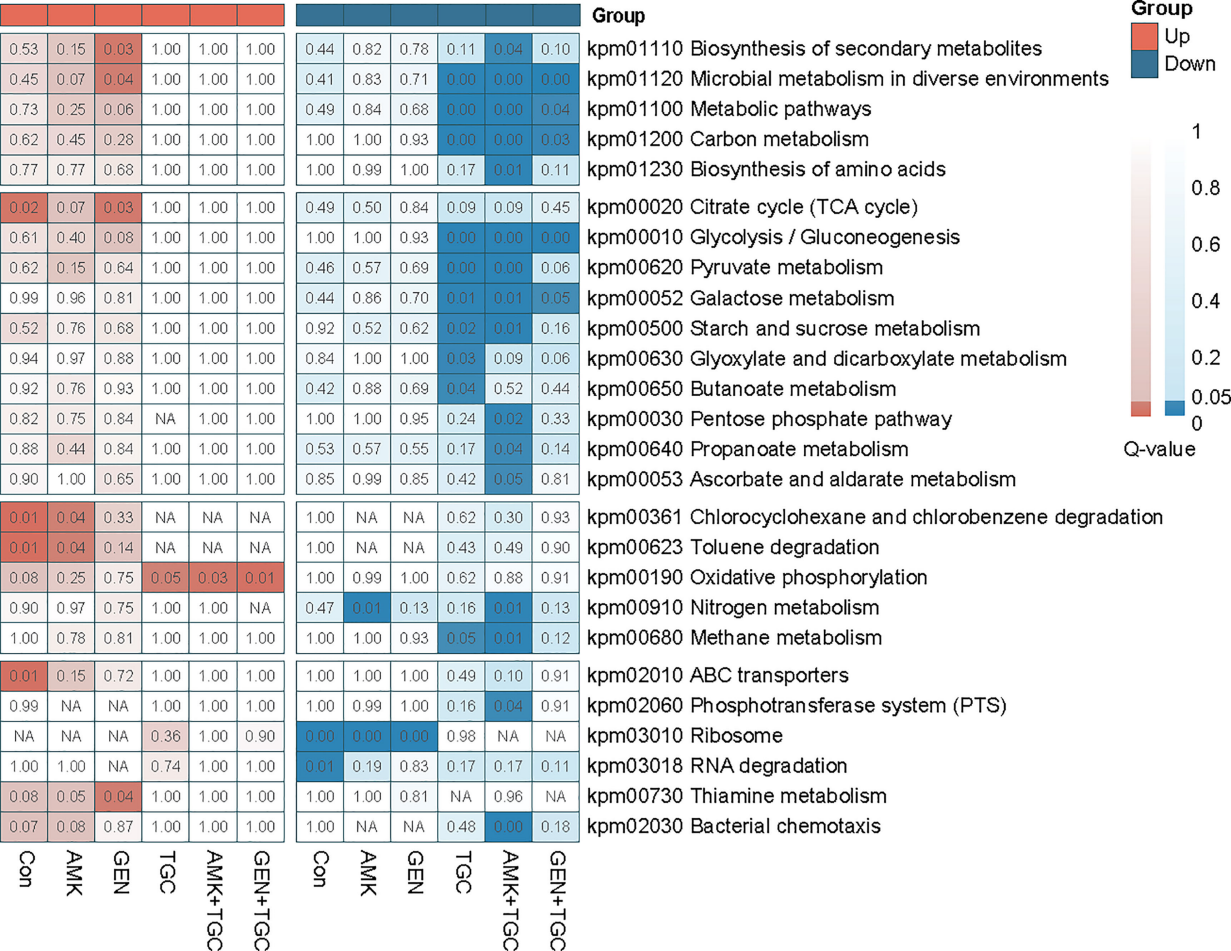
Q-value heatmap of KEGG enrichment analysis of upregulated and downregulated proteins from antibiotic monotherapy and combination therapy. Deeper coloration indicates more significant enrichment. NA, Not Applicable.

**Figure 4:**
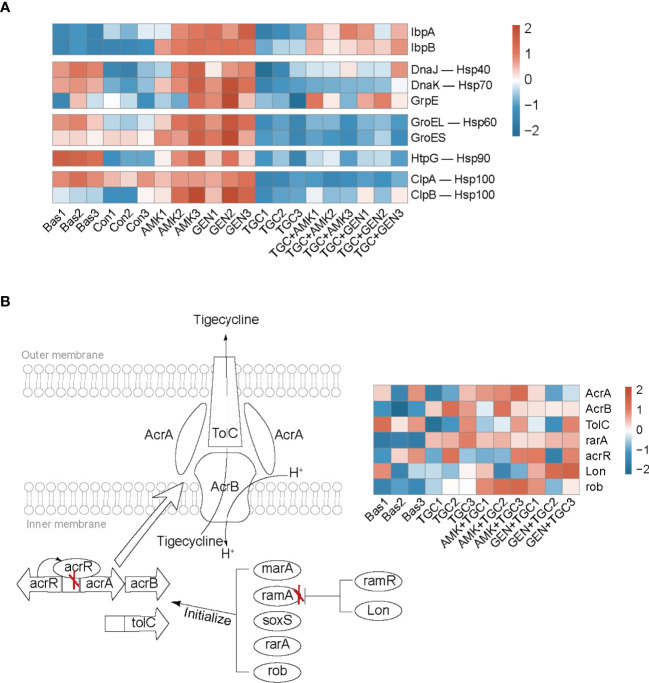
Bacterial adaptive responses to aminoglycosides and tigecycline. **(A)** Heatmap profile of the relative abundance of proteins involved in heat shock response. **(B)** Mechanism diagram of tigecycline resistance based on the regulation of the efflux pump AcrAB-TolC, and the red “X” represents the effect of inhibition. The heatmap profile of the relative abundance of proteins involved in tigecycline resistance. Bas, baseline; Con, control; TGC, tigecycline; AMK, amikacin; GEN, gentamicin.

The bacterial strains in the control group gradually entered the stationary phase after 2 h incubation of baseline bacteria. The highly enriched and downregulated KEGG pathways in the control group included ribosome and RNA degradation (**Figure 3**). In addition, translation, peptide metabolic process and carbohydrate metabolic process were also significantly downregulated (**Figure S1**). The upregulated biological processes included cellular response to environmental stimuli and abiotic stimuli, as well as those involved in the periplasmic space and ligand-gated channel activity (**Figure S2**). The proteomic responses of bacteria exposed to amikacin and gentamicin were generally similar to those of the control group, with downregulation of ribosomes, translation and peptide metabolic process observed in both groups (**Figures 3** and **S1**). Besides, heat shock proteins such as IbpA, IbpB, DnaK, and GroEL were upregulated in bacteria exposed to aminoglycosides in comparison with the control group (**Figure 4A**).

Tigecycline induced distinct bacterial responses as compared to that observed in response to aminoglycosides. We observed significant downregulation of carbohydrate metabolism in tigecycline-treated strains, including downregulated glycolysis/gluconeogenesis, pyruvate metabolism, and starch and sucrose metabolism (**Figure 3**). Notably, oxidative phosphorylation was significantly activated in tigecycline-treated strains, including the upregulation of NADH dehydrogenase and the proton-transporting ATP synthase complex (**Figures 3** and **S2**). Overexpression of the efflux pump is the most significant mechanism that confers tigecycline resistance (Pournaras et al., 2016), and oxidative phosphorylation is a process coupled with the formation of the proton-motive force (PMF), which can facilitate the pumping of tigecycline. As shown in **Figure 4B**, we also observed upregulated expression of *acrB, rarA* and *rob* after 2 h of exposure, while the expression of *acrA* and *tolC* varies in different replicates (**Table S2**). Other upregulated proteins were enriched in regulation of developmental processes and cellular macromolecule metabolic processes, representing the potential adaptive responses of bacteria (**Figure S2**).

### Proteomic Responses to Combination Therapy Were Dominated by Tigecycline

According to the proteomics analysis, tigecycline dominated the proteomic responses in the combination group, and downregulated carbohydrate metabolism was identified in both the tigecycline and drug-combination groups (**Figures 3** and **S1**). Upregulation of the oxidative phosphorylation, NADH dehydrogenase and ribosomal proteins was also observed in both antibiotic combination groups (**Figures 3** and **S3**). Furthermore, expression of heat shock proteins was inhibited in drug-combination groups (**Figure 4A**). In combination therapy with tigecycline and amikacin, 98 DEPs were uniquely upregulated, and 86 DEPs were uniquely downregulated. A total of 44 DEPs were uniquely upregulated in the tigecycline combined with gentamicin, and 46 DEPs were uniquely downregulated (**Figure S4**).

### The Rapid Emergence of Resistance After Exposure to Tigecycline in CRKP

**Figure 5:**
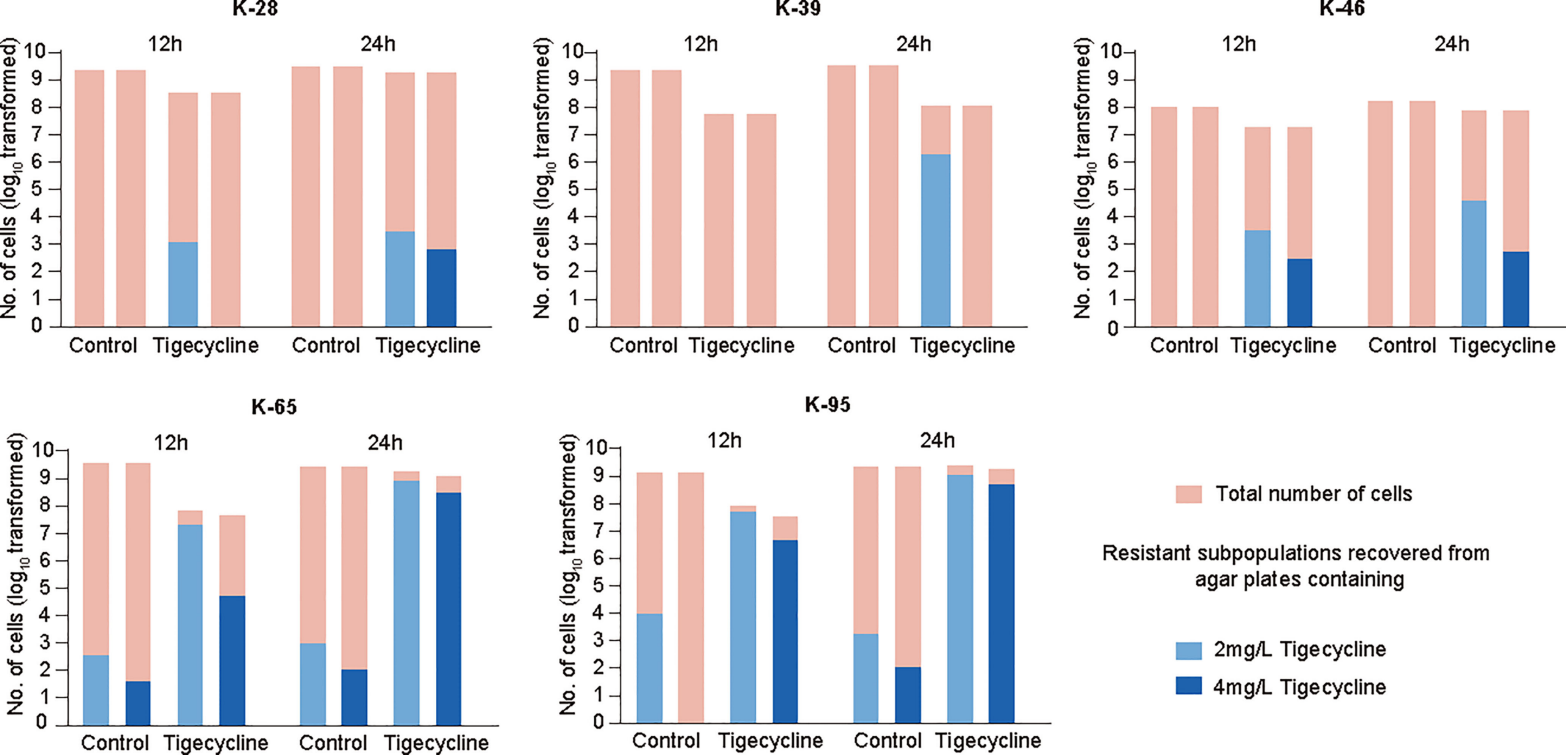
Emergence of resistant strains after tigecycline exposure and analysis of gene expression. The number of resistant subpopulations at 12 h and 24 h in time-kill assays. Representative isolates with heteroresistance (K-65 and K-95) and without heteroresistance (K-28, K-39, and K-46) were selected.

The proteomics results indicated that CRKP can rapidly evolved adaptive responses to tigecycline, possibly indicating the evolution of resistance. Here we further explored the occurrence of drug resistance of CRKP after short-term exposure to tigecycline. The extensive emergence of resistance can be due to either a selection of pre-existing resistant subpopulations or adaptations of sensitive populations. To explore this question for tigecycline, we first evaluated the prevalence of tigecycline heteroresistance in CRKP isolates. Heteroresistance can be defined as the presence of a heterogeneous population of bacteria that exhibit increased levels of antibiotic resistance as compared with the main population, which may be associated with the rapid emergence of resistance after treatment (Andersson et al., 2019). Among 100 non-duplicate CRKP clinical isolates, 43 showed tigecycline heteroresistance, two showed resistance, and 10 showed intermediate resistance (**Figure S5A**). Strain K-28 used in the proteomic investigation did not exhibit heteroresistance. We then selected two other strains with heteroresistance and two strains without heteroresistance for further analysis. In general, there was no significant difference in the time-kill curves among the strains (**Figure S5B**). To explore the possible emergence of antibiotic-resistant strains, we measured the resistant subpopulations recovered from agar plates containing 2 mg/L or 4 mg/L tigecycline after 12 h and 24 h of treatment. The results demonstrated that continuous exposure to tigecycline offered a significant selective effect on heterogeneous subpopulations in strains with heteroresistance (K-65 and K-95) (**Figure 5**). It is worth noting that we also observed the emergence of resistance in strains without heteroresistance (K-28, K-39, and K-46) (**Figure 5**), suggesting that the adaptation of sensitive strains eventually evolve into stable resistant strains over time.

### Evolutionary Hypersensitivity to Aminoglycosides in Tigecycline-Resistant Strains

**Figure 6:**
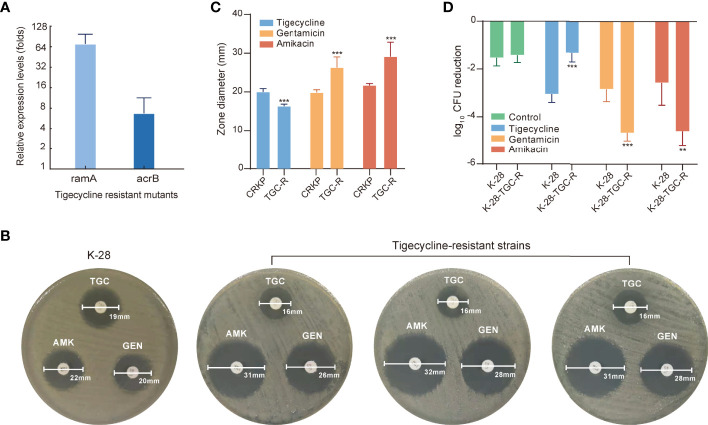
Evolutionary hypersensitivity to aminoglycosides in tigecycline-resistant strains. **(A)** Expression levels of *ramA* and *acrB* were determined in tigecycline-resistant strains using qRT–PCR. Error bars indicate SDs. **(B)** The antibiotic susceptibility of K-28 and representative tigecycline-resistant strains measured by disk-diffusion susceptibility testing. **(C)** Bar plot of the zone diameter in disk-diffusion susceptibility tests for tigecycline-susceptible and -resistant strains. **(D)** *In vivo* antimicrobial efficacy of tigecycline or aminoglycosides for treating K-28 and a tigecycline-resistant strain derived from K-28 and using rat tissue cage infection models. Error bars indicate SDs. **P < 0.01; ***P < 0.001. TGC, tigecycline; TGC-R, tigecycline resistant; AMK, amikacin; GEN, gentamicin.

Tigecycline-resistant strains were used for further analysis, with significant upregulation of *ramA* and *acrB* expression detected in all tigecycline-resistant strains according to quantitative reverse transcription (qRT)-PCR (**Figure 6A**). Proteomics results suggested that the adaptive evolution make tigecycline-sensitive strains more prone to be more susceptible to aminoglycosides. We further explored whether tigecycline-resistant strains were also more susceptible to aminoglycosides by using the disk-diffusion method. **Figure 6B** shows the zone diameters of the K-28 and representative tigecycline-resistant strains. The zone diameter to aminoglycosides was significantly increased in tigecycline-resistant strains, with subsequent testing of more CR-KP and their tigecycline-resistant strains confirming these results (**Figure 6C**). To determine the clinical relevance of the increased sensitivity to aminoglycosides in tigecycline-resistant strains *in vivo*, we tested the ability of aminoglycosides to treat infection with tigecycline-resistant strains in rat tissue cage infection models. Animal experiments further confirmed the results of *in vitro* studies, with the therapeutic effect of aminoglycosides on the tigecycline-resistant strain better than that on the tigecycline-sensitive strain K-28 (**Figure 6D**).

## Discussion

Tigecycline and aminoglycosides have been used for the treatment of CRO infections as two major classes of antibiotics (Peterson, 2008; Jackson et al., 2013). In the present study, we used CRKP to explore the bacterial response to these two antibiotics. Proteomics analysis revealed that tigecycline and aminoglycosides induced diverse or even opposite bacterial responses. Although aminoglycosides bind to the decoding center of the ribosome, this does not translate to an immediate standstill of ribosomal activity. Instead, their binding promotes protein mistranslation through the incorporation of inappropriate amino acids (Aguirre Rivera et al., 2021; Wohlgemuth et al., 2021). Aminoglycoside-mediated killing has been linked to alterations in the cell-membrane ultrastructure (Anand et al., 1960; Anand and Davis, 1960; Davis et al., 1986; Baquero and Levin, 2021), and the bactericidal effect of aminoglycosides occurs quickly with the accumulation of mistranslated proteins (Davies et al., 1965; Wohlgemuth et al., 2021). Bacterial mistranslated proteins can lead to misfolding, which induces the heat shock response to reduce the damage (Ling et al., 2012). We noticed significant upregulation of IbpA and IbpB in bacteria exposed to aminoglycosides, similar to previous studies (Wu et al., 2015; Wohlgemuth et al., 2021). Compared with control group, the expression of chaperone proteins such as GroEL/GroES and DnaK/DnaJ/GrpE was slightly higher in bacteria exposed to aminoglycosides, and associated with the short-term tolerance to aminoglycosides (Goltermann et al., 2013). Downregulation of ribosomal proteins and translation inhibition could be another adaptative response of bacteria and are associated with antibiotic resistance (Cho et al., 2015; Coleman Shannon et al., 2020), which might mitigate the protein mistranslation through reducing the aminoglycoside binding sites.

In contrast to aminoglycosides with bactericidal effects, tigecycline with bacteriostatic effects only interferes with cellular growth and metabolism and is insufficient to induce cell death. According to the time-kill assays, there was no significant reduction in cell populations after 2 h of tigecycline exposure. The proteomics data collected during this period mainly reflected drug effects and a series of adaptive bacterial responses. The non-bactericidal effect of tigecycline offered sufficient time for strains to adjust their growth and metabolic state to minimize the impact of the drugs. To rescue the stalled translation process, bacteria evolve to induce the extensive upregulation of ribosomal and translation-related proteins, with similar responses previously identified in *Escherichia coli* to cope with chlortetracycline stress (Lin et al., 2014). In addition to direct compensation for translation inhibition, bacteria have evolved enhanced antibiotic efflux pumps to reduce intracellular drug concentrations (Pournaras et al., 2016). Moreover, activation of oxidative phosphorylation and the subsequent proton electrochemical gradient facilitates drugs being pumped out of cells (Seeger et al., 2006; He et al., 2015).

**Figure 7:**
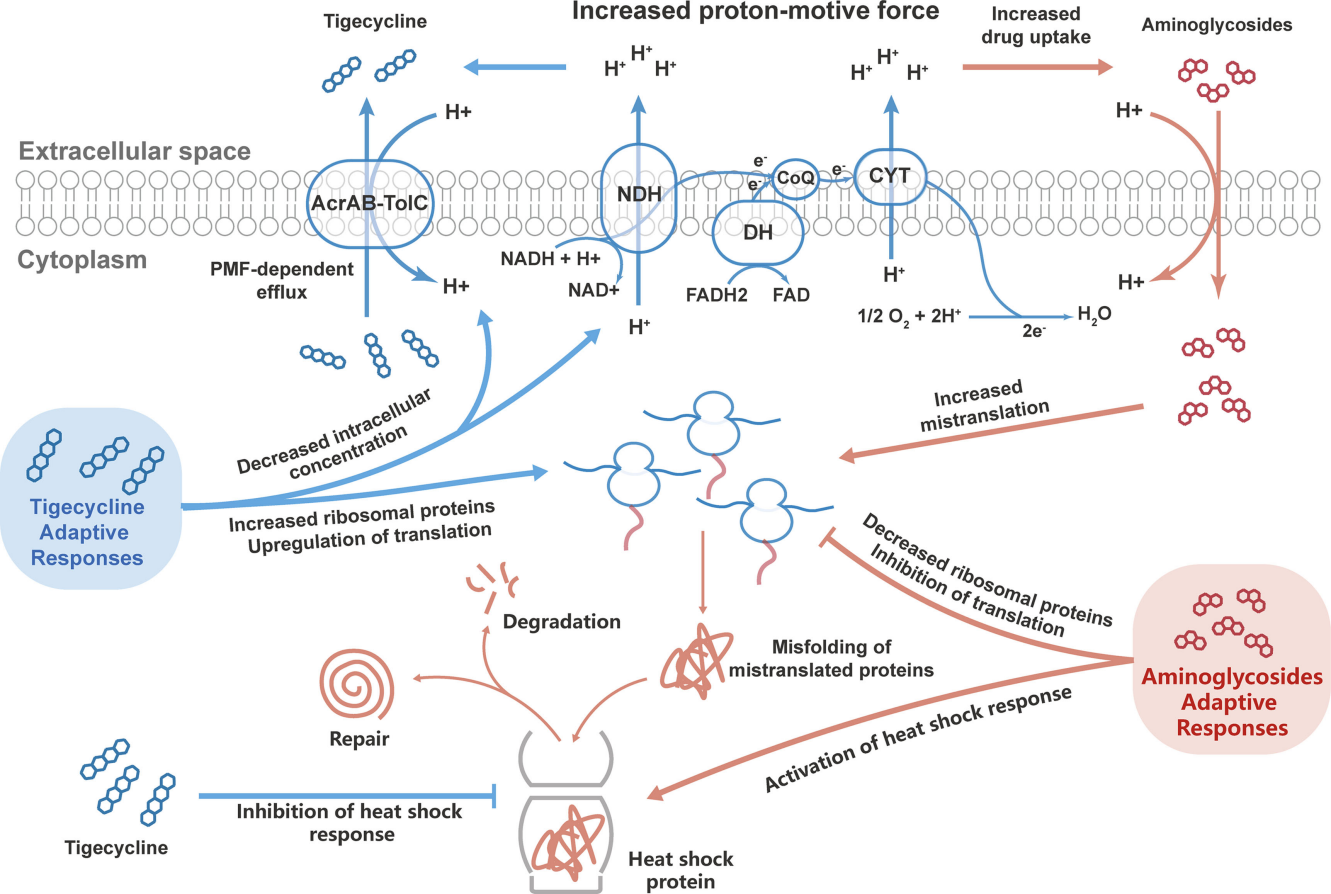
Potential mechanisms of the synergistic effects of tigecycline combined with aminoglycosides against CRKP. Bacteria can develop adaptive responses to tigecycline and aminoglycosides. During combination therapy, tigecycline dominated the bacterial responses, which might lead to synergistic bactericidal effects by enhancing the aminoglycoside effect and inhibiting the adaptive responses of bacteria to aminoglycosides.

Intriguingly, bacteriostatic–bactericidal combination treatments show antagonism in most cases, indicating that the bactericidal activity is attenuated in combination therapy (Ocampo et al., 2014; Lobritz et al., 2015; Stokes et al., 2019). In general, during bacteriostatic–bactericidal combination treatments, the phenotypic outcome is dominated by bacteriostatic antibiotic, resulting in suppressed cellular respiration and diminished metabolic flux, thereby blocking bactericidal killing (Ocampo et al., 2014; Lobritz et al., 2015). In the present study, the proteomic responses in the combination group were dominated by tigecycline as well, and downregulated carbohydrate metabolism was identified in both the tigecycline and drug-combination groups, suggesting a potential antagonism between tigecycline and aminoglycosides. How to explain the synergistic effects of antibiotic combinations? Based on proteomics research, the bacterial adaptative responses might play important roles. Here we proposed the following mechanisms for the synergism of tigecycline and aminoglycosides (**Figure 7**). In combination therapy, tigecycline dominated the response, and the adaptive evolution of tigecycline increased generation of the PMF, which might enhance the activity of the AcrAB-TolC efflux pump but in turn promoted the uptake of aminoglycosides through PMF-dependent process. Moreover, tigecycline perturbed bacterial adaptive responses to aminoglycosides during combination therapy through upregulating ribosomal proteins and inhibiting heat shock response. In general, the combination of the two drugs created a dilemma for CRKP and ultimately generated a synergistic effect.

The above mechanisms derived from the proteomics analysis require further validation. However, capturing the susceptibility changes at single-cell levels in CRKP after short adaptive responses to tigecycline is quite hard. In general, bacteria have to respond to antibiotic environment for surviving, and ultimately evolves resistance. Therefore, we further analyzed bacterial evolution after exposure to tigecycline and evaluated the susceptibility changes in tigecycline-resistant strains. We identified that tigecycline-resistant strains can emerge within 24h of exposure in clinical isolates without heteroresistance. In line with proteomic findings, we observed that tigecycline-resistant strains were more susceptible to aminoglycosides using *in vitro* and *in vivo* assays. These results confirmed that adaptation to tigecycline made CRKP more susceptible to aminoglycosides.

This study has some limitations, firstly, the proteomics experiment was conduct in only one strain, and the sample size needs to be expended in the future research. Secondly, the synergetic mechanisms were mainly drawn from the proteomics analysis, which required further validation.

## Conclusions

Antibiotic combination therapies have been intensively studied in recent decades, and increasing antibacterial effects and inhibiting resistance occurrence have been regarded as essential issues in this field. Through proteomics analysis, we identified that proteomic responses to tigecycline and aminoglycosides were divergent in monotherapy, and proteomic alterations to combination therapy were dominated by tigecycline. Moreover, we demonstrated that adaptive response to tigecycline in CRKP altered the sensitivity to aminoglycosides, which might be associated with the synergistic effect of combination therapy with the two drugs. Overall, these findings provide novel insight into antibiotic synergetic mechanisms based on the evolution of bacteria under antimicrobial selection pressure, which deserves more attention in future on designing antibiotic combination therapy regimens.

## Data Availability Statement

The datasets presented in this study can be found in online repositories. The names of the repository/repositories and accession number(s) can be found in the article/**Supplementary Material**

## Ethics Statement

The animal study was reviewed and approved by Institutional Animal Care and Use Committee of Peking University People’s Hospital.

## Author Contributions

XM, SF, and YW: Data curation, Formal analysis, Investigation, Methodology, Project administration and Writing - original draft. LZ, WY, and YH: Data curation, Formal analysis, and Methodology. WN: Conceptualization, Funding Acquisition, Data curation, Formal analysis, Supervision and Writing - review and editing. ZG: Conceptualization, Formal analysis, Supervision and Writing - review and editing. All authors contributed to the article and approved the submitted version.

## Funding

This work was supported by the National Natural Science Foundation of China (81903672), China International Medical Foundation (Z-2018-35-2003), and Peking University People’s Hospital Research and Development Funds (RS2020-04).

## Conflict of Interest

The authors declare that the research was conducted in the absence of any commercial or financial relationships that could be construed as a potential conflict of interest.

## Publisher’s Note

All claims expressed in this article are solely those of the authors and do not necessarily represent those of their affiliated organizations, or those of the publisher, the editors and the reviewers. Any product that may be evaluated in this article, or claim that may be made by its manufacturer, is not guaranteed or endorsed by the publisher.
