# Supplementary material for: Proteomics Study of the Synergistic Killing of Tigecycline in Combination With Aminoglycosides Against Carbapenem-Resistant Klebsiella pneumoniae
Source: Front Cell Infect Microbiol. 2022 Jun 30;12:920761. doi: 10.3389/fcimb.2022.920761 (PMC9280366; doi:10.3389/fcimb.2022.920761)
Supplement: Supplementary file 1 [file Table_1.docx]

**Proteomics Study of the Synergistic Killing of Tigecycline in Combination with Aminoglycosides against Carbapenem-resistant Klebsiella pneumoniae**

**Running title:** Proteomics study in combination therapy

**Author:** Xinqian Ma^†^, Shining Fu^†^, Yifan Wang, Lili Zhao, Wenyi Yu, Yukun He, Wentao Ni^*^, Zhancheng Gao^*^

**Institutional affiliations:**

Department of Pulmonary and Critical Care Medicine, Peking University People’s Hospital, Beijing, China

† These authors equally contributed to this article.

* Corresponding author:

Wentao Ni, Email: wentao.qingdao@163.com, Department of Pulmonary and Critical Care Medicine, Peking University People’s Hospital, Beijing, China;

Zhancheng Gao, Email: zcgao@bjmu.edu.cn, Department of Pulmonary and Critical Care Medicine, Peking University People’s Hospital, Beijing, China.

**Table of contents**

[**Supplementary Texts** 3](#_Toc103539396)

[**Text S1** Proteomics analysis 3](#_Toc103539397)

[**Supplementary Figures** 6](#_Toc103539398)

[**FIG S1** Q-value heatmap of downregulated proteins based on GO enrichment analysis 6](#_Toc103539399)

[**FIG S2** Q-value heatmap of upregulated proteins based on GO enrichment analysis 7](#_Toc103539400)

[**FIG S3** Distinct proteomic response induced by different therapeutic strategies 8](#_Toc103539401)

[**FIG S4** Venn diagram of differentially expressed proteins (DEPs) between groups 9](#_Toc103539402)

[**FIG S5** Susceptibility profile and time–kill assay of CRKP 10](#_Toc103539403)

[**Supplementary Tables** 11](#_Toc103539404)

[**Table S1** Primers used for qRT-PCR analysis 11](#_Toc103539405)

[**Table S2** Expression of tigecycline resistance-related proteins (TGC vs Bas) 11](#_Toc103539406)

[**Table S3** Elution gradient of UPLC 12](#_Toc103539407)

[**Table S4** Liquid chromatography gradient of EASY-nLC 1200 13](#_Toc103539408)

# **Supplementary Texts**

# **Text S1** Proteomics analysis

***Protein extraction and sample preparation***

Bacterial samples were centrifuged at 5,000 rpm for 15 min at 4°C, washed three times with 1× phosphate-buffered saline, and then resuspended in protein lysis buffer (8 M urea, 1% sodium dodecyl sulfate, and protease inhibitor). Sonicated suspension was incubated on ice for 30 min with vortexing for 5 s to 10 s every 5 min. The mixture was then centrifuged at 4°C and 16,000 ×g for 30 min. The supernatant was transferred into a new tube, and protein concentrations were quantified using a Pierce BCA Protein Assay Kit BCA (Thermo Fisher Scientific, Waltham, MA, USA). For proteolysis, the total protein (100 μg) of each sample was added with triethylammonium bicarbonate buffer to a final concentration of 100mM (pH = 7~8), the mixture was reduced with Tris (2-carboxyethyl) phosphine to a final concentration of 10mM at 37°C for 1 h, and alkylated with iodoacetamide to a final concentration of 40mM for 40 min in the dark at room temperature. After centrifugation at 4°C and 10,000 ×g for 20 min, the precipitates were collected and resuspended in 100 μL 100 mM triethylammonium bicarbonate buffer. Trypsin was added at a protein:trypsin ratio of 50:1 to digest the proteins overnight at 37°C.

***Liquid chromatography-tandem mass spectrometry (LC-MS/MS) analysis***

Data-dependent acquisition (DDA) mode was used to build a spectra library. Equal amounts of trypsin-digested peptides of each sample were pooled together, dried under vacuum, and resuspended in buffer A [2% acetonitrile (ACN), pH 10.0, adjusted with ammonium hydroxide]. The mixture was fractionated by Vanquish Flex ultra-high-performance LC (Thermo Fisher Scientific, Waltham, MA, USA) with an ACQUITY UPLC BEH C18 column (1.7 µm, 2.1 mm × 150 mm; Waters Corp., Milford, MA, USA). Briefly, peptides were first separated with a gradient of elution (buffer B: 80% ACN, pH 10.0, adjusted with ammonium hydroxide) over 47 min at a flow rate of 200 μL/min. The elution gradient is shown in Table S3. Twenty fractions were collected and dried under vacuum. The peptides were redissolved in solvent A (2% ACN with 0.1% formic acid) with appropriate 10× indexed retention time (iRT) peptide, and analyzed by an on-line nanoelectrospray Q Exactive HF-X quadrupole Orbitrap mass spectrometer (Thermo Fisher Scientific, Waltham, MA, USA) coupled with an EASY-nLC 1200 system (Thermo Fisher Scientific, Waltham, MA, USA). Briefly, a C18 column (75 μm × 25 cm; Thermo Fisher Scientific) was equilibrated with solvent A and solvent B (B: 80% ACN with 0.1% formic acid). The elution gradients are shown in Table S4, and the column flow rate was maintained at 300 nL/min. The MS scan was performed as follows: scan range (m/z) = 300–1500, resolution = 60,000, automatic gain control (AGC) target = 3e6, maximum injection time = 20 ms. The high-energy collision dissociation (HCD) MS/MS scan: resolution = 15,000, AGC target = 5e4, maximum injection time = 45 ms.

The individual samples of each group were then analyzed in data-independent acquisition (DIA) mode. The full-scan MS spectra (m/z 300–1500) were acquired with a resolution of 60,000. All of the precursor ions were fragmented by HCD for MS/MS scan. DIA was performed with 40 variable isolation windows. The LC gradient conditions and other MS parameters were the same as DDA mode.

***Protein identification and statistical analyses***

The DDA data were analyzed by ProteomeDiscoverer (Version 2.4; Thermo Fisher Scientific, Waltham, MA, USA) with the default settings. Carbamidomethyl was specified as the static modification, oxidation and acetylation (protein N-terminus) were specified as dynamic modifications. The false discovery rate (FDR) was set to 1% at both the peptide and protein levels. The DIA data were analyzed using Spectronaut (Version 14.0; Biognosys AG, Schlieren, Switzerland). The retention time prediction type was set to dynamic iRT. Data extraction was performed using Spectronaut (Version 14.0; Biognosys AG, Schlieren, Switzerland) based on extensive mass calibration. Q-value cutoff of peptide and protein level was set to 1%, and quantification was performed using the top 6 filtered peptides. A total of 2,873 proteins were identified as belonging to the proteome of K. pneumoniae. Differentially expressed proteins (DEPs) were identified according to a fold change (FC) >1.2 or <0.83 and an adjusted P < 0.05. DEPs were further used for Gene Ontology (GO) and Kyoto Encyclopedia of Genes and Genomes (KEGG) enrichment analyses, and P values were adjusted by Benjamini-Hochberg method.

# **Supplementary Figures**

# **FIG S1** Q-value heatmap of downregulated proteins based on GO enrichment analysis

Q-value heatmap of top 15 Gene Ontology (GO) enrichment analysis of downregulated proteins from antibiotic monotherapy and combination therapy. Deeper coloration indicates more significant enrichment. Con, control; TGC, tigecycline; AMK, amikacin; GEN, gentamicin.


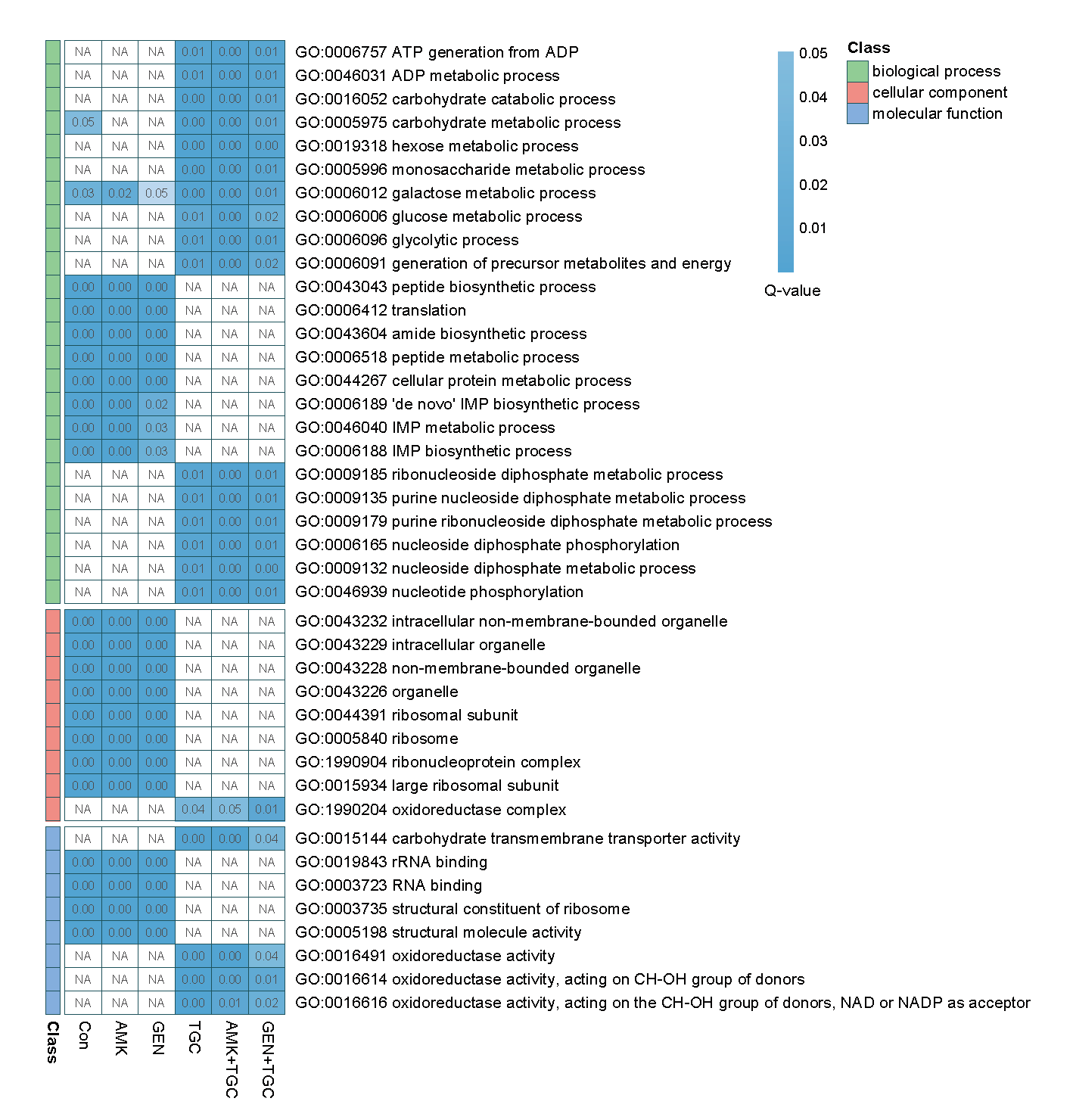


# **FIG S2** Q-value heatmap of upregulated proteins based on GO enrichment analysis

Q-value heatmap of top 15 Gene Ontology (GO) enrichment analysis of upregulated proteins from antibiotic monotherapy and combination therapy. Deeper coloration indicates more significant enrichment. Con, control; TGC, tigecycline; AMK, amikacin; GEN, gentamicin.


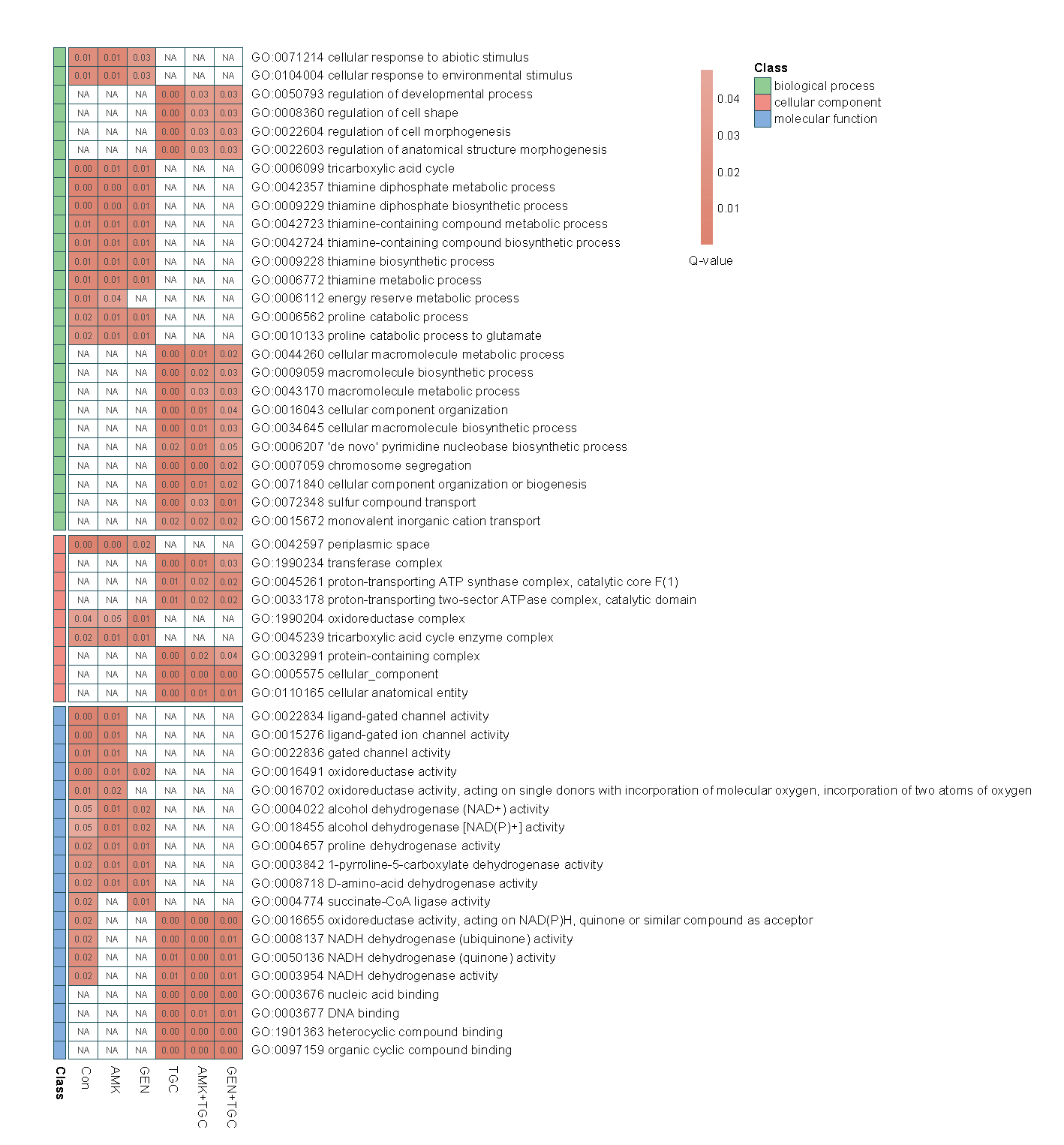


# **FIG S3** Distinct proteomic response induced by different therapeutic strategies

(A) Heatmap profile of relative abundance of ribosomal proteins. (B) Heatmap profile of relative abundance of translation regulators. (C) Heatmap profile of relative abundance of proteins involved in oxidative phosphorylation. Bas, baseline; Con, control; TGC, tigecycline; AMK, amikacin; GEN, gentamicin.


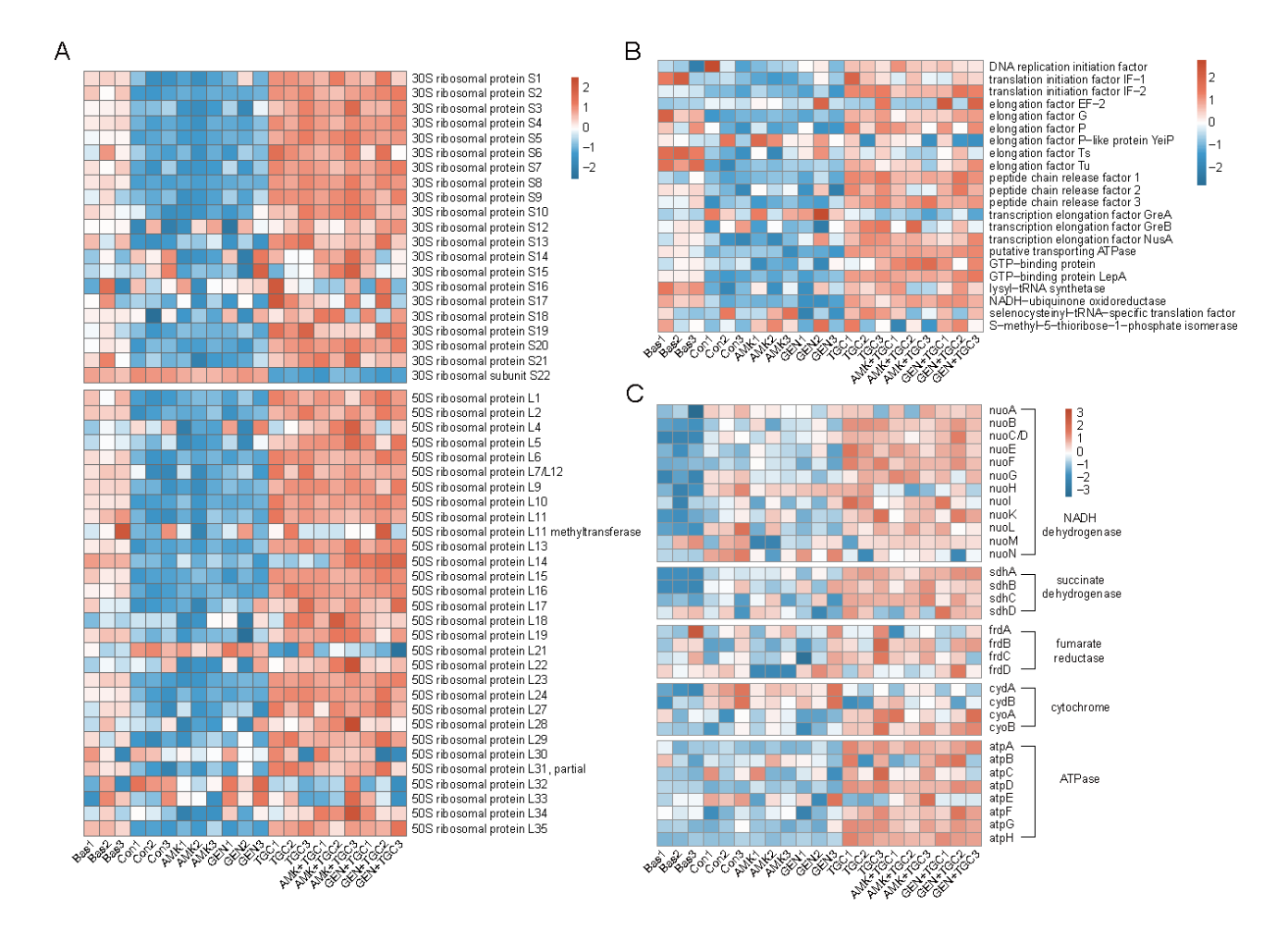


# **FIG S4** Venn diagram of differentially expressed proteins (DEPs) between groups

(A, B) Venn diagram of up-regulated and down-regulated DEPs in amikacin and tigecycline monotherapy and combination therapy. (C, D) Venn diagram of up-regulated and down-regulated DEPs in gentamicin and tigecycline monotherapy and combination therapy.


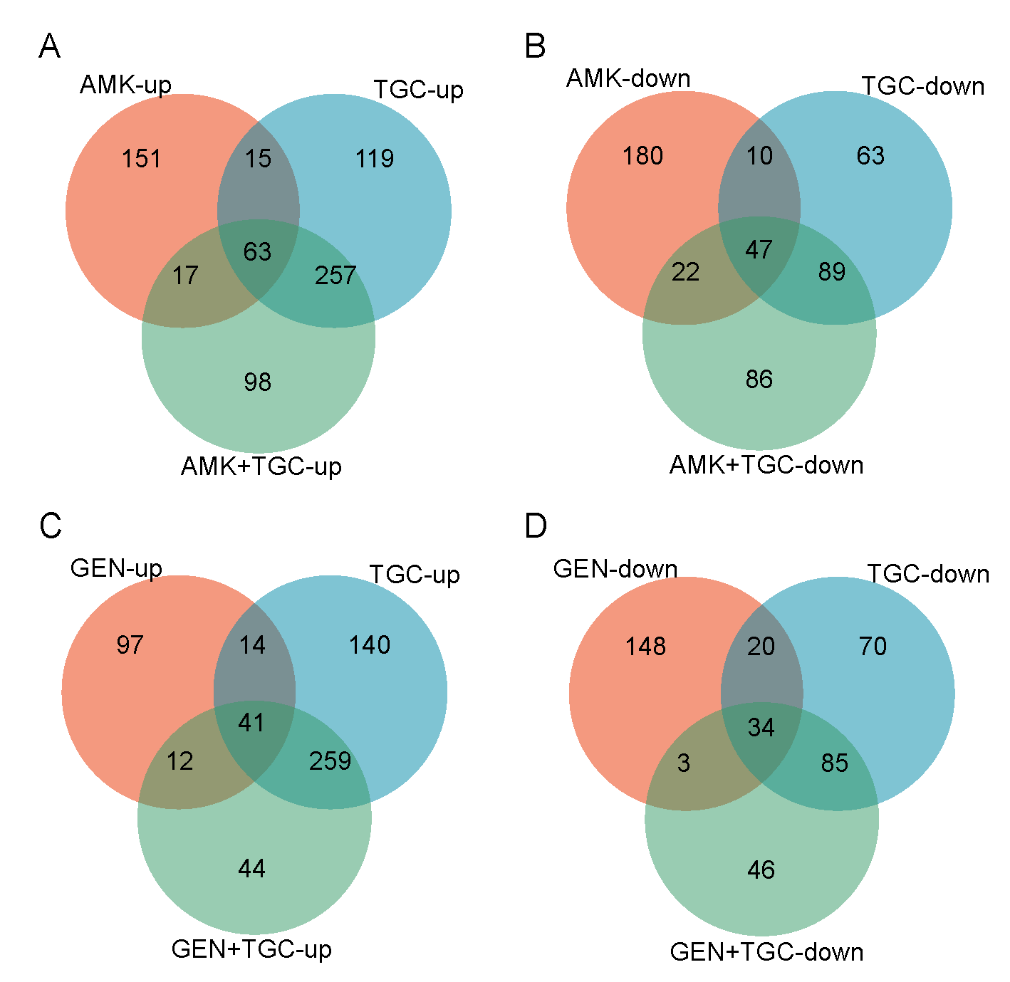


# **FIG S5** Susceptibility profile and time–kill assay of CRKP

(A) Distribution of the sensitivity and heteroresistance to tigecycline in 100 CRKP clinical isolates. (B) Time-kill curves of strains with tigecycline heteroresistance (K-65 and K-95) and without heteroresistance (K-28, K-39, and K-46). Con, control; TGC, tigecycline.

**
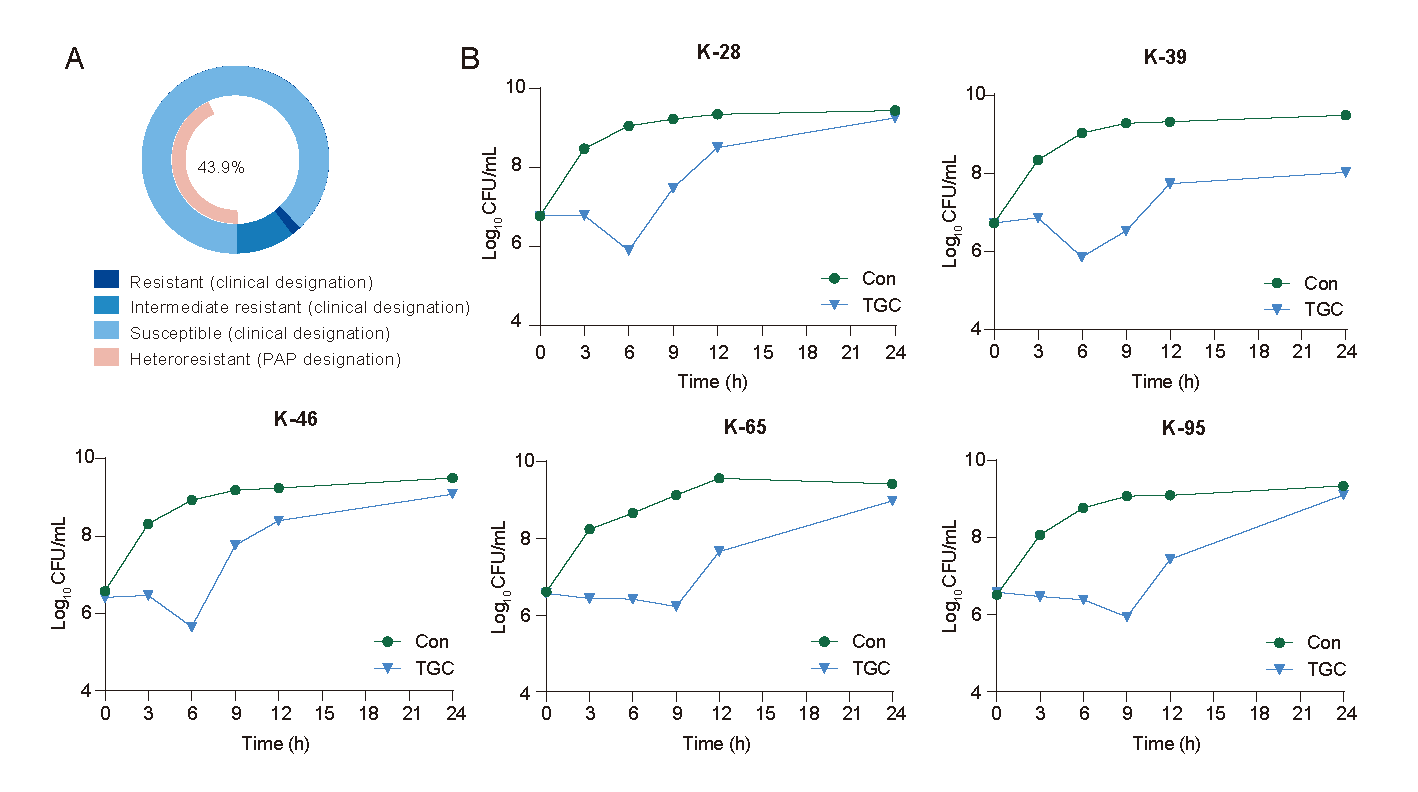
**

# **Supplementary Tables**

# **Table S1** Primers used for qRT-PCR analysis

| Gene | Sequence |
| --- | --- |
| *ramA*-F | GATATCGCTCGCCATGC |
| *ramA*-R | CTGTGGTTCTCTTTGCGGTAG |
| *acrB*-F | AAACTTCGCCACTACGTCATA |
| *acrB*-R | AGCTTAACGCCTCGATCAT |
| *rrsE*-F | GTCATCATGGCCCTTACGAG |
| *rrsE*-R | ACTTTATGAGGTCCGCTTGCT |

# **Table S2** Expression of tigecycline resistance-related proteins (TGC vs Bas)

| Protein | FC | Padj |
| --- | --- | --- |
| acrA | 0.9794 | 0.6907 |
| acrB | 1.293 | 0.04426 |
| tolC | 0.8025 | 0.3194 |
| acrR | 1.293 | 0.9489 |
| rarA | 1.815 | 0.006274 |
| rob | 1.424 | 0.03603 |
| Lon | 0.998 | 0.9946 |

Bas, baseline; TGC, tigecycline.

# **Table S3** Elution gradient of UPLC

| Time (min) | B (%) |
| --- | --- |
| 0 | 0 |
| 16 | 0 |
| 17 | 3.8 |
| 34 | 24 |
| 37 | 30 |
| 38 | 43 |
| 39 | 100 |
| 44 | 0 |
| 47 | 0 |

# **Table S4** Liquid chromatography gradient of EASY-nLC 1200

| Time (min) | B (%) |
| --- | --- |
| 0 | 5 |
| 70 | 23 |
| 90 | 29 |
| 100 | 38 |
| 102 | 48 |
| 103 | 100 |
| 110 | 100 |
| 120 | STOP |
